# BrainFusion: a Low‐Code, Reproducible, and Deployable Software Framework for Multimodal Brain‒Computer Interface and Brain‒Body Interaction Research

**DOI:** 10.1002/advs.202417408

**Published:** 2025-06-05

**Authors:** Wenhao Li, Chenyang Gao, Zhaobo Li, Yunheng Diao, Jiaxin Li, Jiayi Zhou, Jing Zhou, Ying Peng, Guanchu Chen, Xuecheng Wu, Kai Wu

**Affiliations:** ^1^ School of Biomedical Science and Engineering South China University of Technology Guangzhou 511442 China; ^2^ School of Material Science and Engineering South China University of Technology Guangzhou 510006 China; ^3^ National Engineering Research Center for Tissue Restoration and Reconstruction South China University of Technology Guangzhou 510006 China; ^4^ Department of Aging Research and Geriatric Medicine Institute of Development Aging and Cancer Tohoku University Sendai 980–8575 Japan

**Keywords:** application deployment, brain‒body interactions, brain‒computer interface, multimodal physiological signals, neurovascular coupling

## Abstract

This study presents BrainFusion, a unified software framework designed to improve reproducibility and support translational applications in multimodal brain–computer interface (BCI) and brain–body interaction research. While ​electroencephalography (EEG)​​‐based BCIs have advanced considerably, integrating multimodal physiological signals remains hindered by analytical complexity, limited standardization, and challenges in real‐world deployment. BrainFusion addresses these gaps through standardized data structures, automated preprocessing pipelines, cross‐modal feature engineering, and integrated machine learning modules. Its application generator further enables streamlined deployment of workflows as standalone executables. Demonstrated in two case studies, BrainFusion achieves 95.5% accuracy in within‐subject EEG–functional near‐infrared spectroscopy (fNIRS)​​ motor imagery classification using ensemble modeling and 80.2% accuracy in EEG–electrocardiography (ECG)​​ sleep staging using deep learning, with the latter successfully deployed as an executable tool. Supporting EEG, fNIRS, electromyography (EMG)​, and ECG, BrainFusion provides a low‐code, visually guided environment, facilitating accessibility and bridging the gap between multimodal research and application in real world.

## Graphical abstract

BrainFusion is a low‐code software framework for multimodal brain–computer interface (BCI)​​ and brain–body interaction research. It supports ​electroencephalography (EEG)​, functional near‐infrared spectroscopy (fNIRS), electromyography (EMG)​, and electrocardiography (ECG)​​ integration with standardized preprocessing, feature fusion, and model deployment. Validated in motor imagery and sleep staging tasks, BrainFusion enhances reproducibility and accelerates translational development in neurophysiological applications.

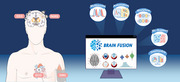

## 1 Introduction

Brain‒computer interface (BCI) technology is a revolutionary technology that enables direct communication between the brain and external devices through the capture and decoding of brain activity signals^[^ ^1^ ^]^ Owing to their noninvasiveness, low cost, and convenience, electroencephalogram (EEG) signals have been used as a primary source of input for BCI research.^[^ ^2^ ^]^ In recent years, EEG‒based BCIs have been extensively developed, and have been applied in various fields, such as neurorehabilitation,^[^ ^3^ ^]^ text input,^[^ ^4^ ^]^ music imagery,^[^ ^5^, ^6^ ^]^ gaming,^[^ ^7^ ^]^ emotion recognition,^[^ ^8^ ^]^ mental fatigue assessment,^[^ ^9^ ^]^ vigilance estimation^[^ ^10^ ^]^ and motion intent recognition.^[^ ^11^ ^]^

EEG analysis software has played a pivotal role in the rapid advancement of EEG‒based BCIs.^[^ ^12^ ^]^ Over the past few decades, EEG analysis tools such as EEGLab,^[^ ^13^ ^]^ FieldTrip,^[^ ^14^ ^]^ BioSig,^[^ ^15^ ^]^ Brainstorm,^[^ ^16^ ^]^ BESA,^[^ ^17^ ^]^ PyEEG,^[^ ^18^ ^]^ and SPM8^[^ ^19^ ^]^ have provided researchers with essential analytical resources, streamlined the EEG analysis workflow, and facilitated communication and data sharing across different laboratories. This has significantly accelerated the progress of BCI research and development. According to Google Scholar, as of July 2024, these analysis tools have been utilized in more than 28000 published studies, and new EEG analysis tools continue to be developed and made available.^[^ ^20^, ^21^, ^22^ ^]^

Single EEG features are often heterogeneous,^[^ ^23^ ^]^ and incorporating physiological signals from other modalities can complement information across signal types, enhancing BCI classification and evaluation.^[^ ^24^, ^25^ ^]^ For example, combining EEG with electrocardiograms (ECG) or galvanic skin responses improves emotion recognition accuracy.^[^ ^24^ ^]^ In complex environments with electromagnetic noise, integrating EEG with functional near‐infrared spectroscopy (fNIRS) enhances reliability.^[^ ^26^, ^27^ ^]^ Additionally, combining EEG with electromyography (EMG) has been shown to improve motor function prediction after strokes,^[^ ^28^ ^]^ and improves classification performances in rehabilitation robotics.^[^ ^29^ ^]^ In response to these developments, many software toolkits have begun to support multimodal signal processing. For example, NeuroKit2^[^ ^30^ ^]^ supports preprocessing and feature extraction for EEG, ECG, and electrooculography (EOG); Brainstorm added an fNIRS plugin in 2016; and the MNE‐Python library^[^ ^31^ ^]^ allows users to analyze EEG, fNIRS, and other signals in a unified environment.

Despite the significant advancements in multimodal physiological signal analysis, several critical challenges impede reproducibility, accessibility, and real‐world applicability. A primary concern is the reproducibility of results, which is often compromised due to variations in data acquisition protocols, preprocessing techniques, and feature extraction parameters. Such inconsistencies can lead to divergent outcomes even when employing identical machine learning or deep learning models. For instance, Rim et al. highlights that the heterogeneity in physiological signal characteristics across studies contributes to inconsistent results in deep learning‐based analyses.^[^ ^32^ ^]^ Similarly, Robbins et al. demonstrate that EEG outcomes are highly sensitive to preprocessing methods, raising concerns about the reliability of published findings.^[^ ^33^ ^]^ Moreover, the lack of publicly available datasets and source code in many deep learning studies further exacerbates reproducibility issues. Roy et al. note that a majority of EEG deep learning studies suffer from poor reproducibility due to the unavailability of their data and code.^[^ ^34^ ^]^ While initiatives like the Mother of All BCI Benchmarks (MOABB) framework have enhanced transparency and reproducibility in EEG‐based deep learning research, similar standardized resources for multimodal physiological data are still lacking.^[^ ^35^ ^]^

The integration of multiple signal modalities also introduces increased complexity in data analysis and algorithm implementation, necessitating advanced programming skills. This complexity poses a barrier for researchers and practitioners without strong computational backgrounds, limiting accessibility and slowing the translation of novel algorithms into practical applications. While many existing tools offer graphical interfaces or script‐based modular workflows, analyses involving complex cross‐modal alignment, coupling computation, and advanced machine learning still often require users to write custom scripts or manually coordinate data across software modules. This creates usability barriers for researchers without extensive programming experience, particularly when dealing with heterogeneous multimodal datasets. Although code‐free and low‐code platforms have been developed in other biomedical domains to address similar challenges, such as the code‐free cloud computing service for biomedical digital signal processing and the Cinco de Bio platform for biomedical imaging research, while comparable tools tailored for multimodal physiological signal analysis remain underdeveloped.^[^ ^36^, ^37^ ^]^

Bridging the gap between laboratory research and real‐world application remains a central challenge in multimodal physiological signal analysis. While algorithmic advances are often demonstrated in controlled experimental settings, their practical deployment requires intermediate steps that translate theoretical models into usable systems. In the field of BCI, this need has been recognized through the development of platforms like OpenVIBE, which was designed to support real‐time BCI prototyping and foster transition toward practical applications.^[^ ^39^ ^]^ Such tools enable iterative testing, user feedback, and integration with real‐world constraints, all of which are essential for developing robust and user‐adapted solutions. However, similar platforms specifically tailored for multimodal physiological signal analysis remain underdeveloped. As Chen et al. emphasize, incorporating usability evaluation and user‐centered design is crucial to ensure the ecological validity of deployed systems.^[^ ^40^ ^]^ Therefore, establishing a reproducible and low‐code software framework that supports prototyping and deployment across multiple modalities would represent a significant step toward closing the gap between research and application in this domain.

To address these challenges, we propose BrainFusion, a unified software framework designed to support both research and translational development in multimodal BCI and brain‒body interaction studies. BrainFusion offers a standardized infrastructure for multimodal data processing, including modality‐specific preprocessing pipelines, a comprehensive library for cross‐domain feature extraction, and a flexible engine for multimodal coupling analysis. It further integrates machine learning and deep learning modules for model benchmarking and uniquely enables the transformation of workflows into deployable applications through its application generator. This paper presents the design rationale, architectural components, and core functionalities of BrainFusion, and demonstrates its practical utility through two representative case studies: EEG‒fNIRS fusion for motor imagery classification and EEG‒ECG integration for deep learning‐based sleep staging. We intend for BrainFusion to complement existing toolkits and contribute to advancing reproducible and accessible multimodal neurophysiological research.

## 2 Experimental Section

### 2.1 Overview of BrainFusion

BrainFusion was a software platform specifically developed to streamline research workflows and accelerate the translation of BCI and brain‒body interaction studies into practical applications. In contrast to existing analytical tools, BrainFusion not only supports comprehensive end‐to‐end pipelines for multimodal physiological signal analysis but also offers integrated solutions for packaging algorithms into standalone executable applications. Its core design philosophy emphasizes low‐code, reproducibility, and deployability, and it was structured around four principal functional modules: a multimodal physiological signal computation engine, a one‐click workflow designer, a machine learning extension framework, and an application generator.

**Figure 1:**
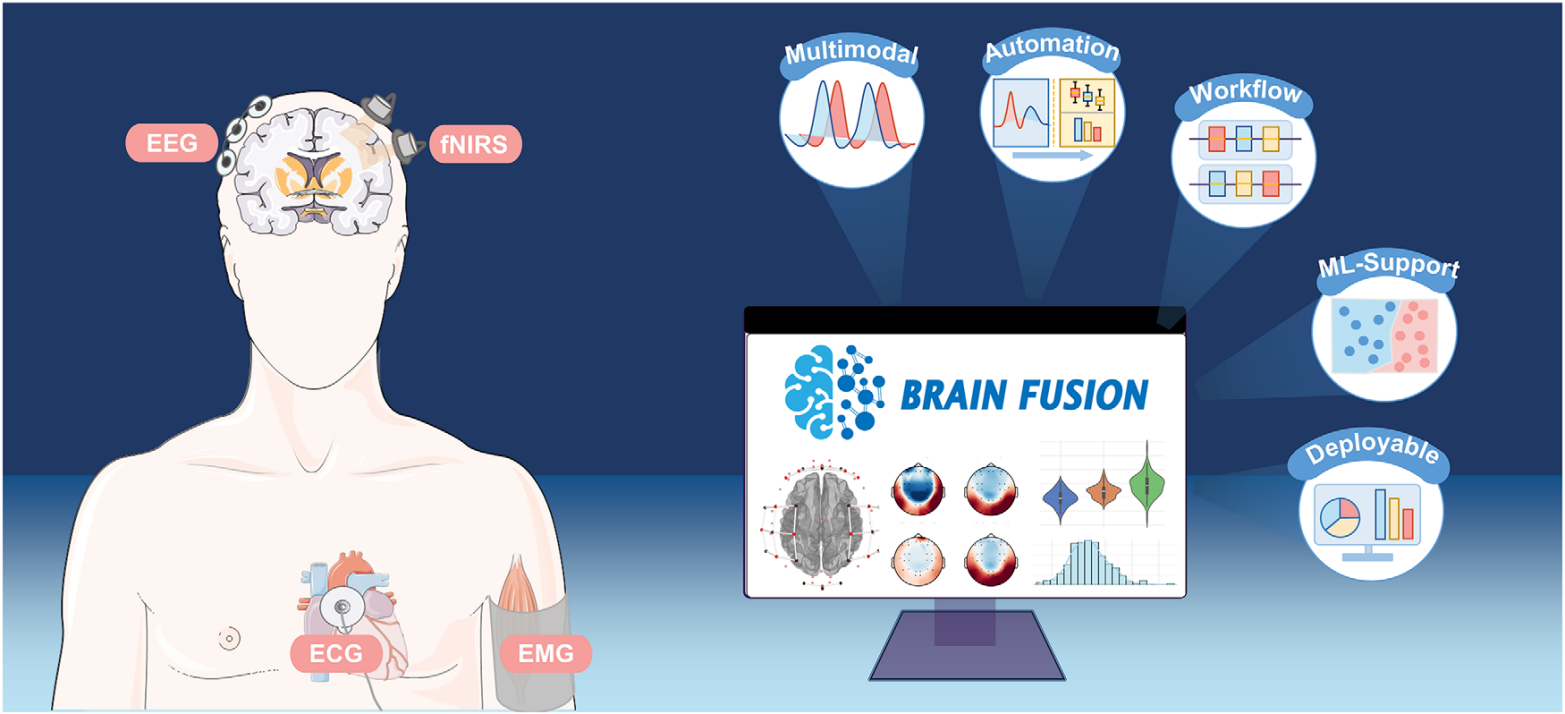
Overview of BrainFusion.

The multimodal physiological signal computation engine provides an extensive computational library for analyzing various physiological parameters, while the one‐click workflow designer enables users to rapidly construct workflows for study replication or customized research development. BrainFusion also incorporates built‐in machine learning algorithms to facilitate feasibility validation, with unique though currently limited support for deep learning models. Additionally, the application generator allows users to export workflows into executable programs for prototype testing or demonstration before product deployment. Currently, BrainFusion provides full‐process support for EEG, fNIRS, EMG, and ECG, with future updates planned to expand compatibility to additional physiological modalities. An overview of the BrainFusion platform function was presented in **Figure** **1**.

### 2.2 Multimodal Physiological Signal Computation Engine

BrainFusion incorporates a multimodal physiological signal computation engine designed for automated batch preprocessing, feature engineering, and coupling analysis across various physiological signal types. It utilizes a standardized data container to store and manage heterogeneous neurophysiological data, including EEG, fNIRS, EMG, and ECG signals. The engine provides validated automatic preprocessing pipelines tailored for each modality, supports comprehensive cross‐domain feature engineering, and implements a dynamic multimodal coupling analysis framework to investigate brain‒body interaction effects.

#### 2.2.1 Standardized Data Container

The multimodal data container in BrainFusion adopts a Python dictionary‐based structure to standardize the storage and management of heterogeneous neural signals originating from diverse sources and formats. Drawing inspiration from the MNE framework, the data structure was categorized into three types: raw, epoch, and evoked. Given the substantial differences in sampling rates and data formats across modalities, BrainFusion incorporates signal alignment during container construction. Two alignment strategies were provided: time‐point alignment for synchronously collected signals and event‐based alignment for asynchronously recorded signals. In time‐point alignment, resampling ensures harmonized sampling rate ratios, whereas in event‐based alignment, alignment was performed based on user‐supplied event files marking event occurrences across signals, with subsequent resampling (Shown in Table S1, Supporting Information). To further enhance interoperability and reproducibility, BrainFusion provides compatibility with the Brain Imaging Data Structure (BIDS)^[^ ^41^ ^]^ format, including support for both EEG‐BIDS^[^ ^42^ ^]^ and NIRS‐BIDS^[^ ^43^ ^]^ specifications, detailing the process of converting raw data into BIDS‐compliant structure in the Supporting Information (Shown in Figure S1, Supporting Information).

#### 2.2.2 Automatic Preprocessing Pipeline

Given the substantial differences in amplitude, noise characteristics, and artifacts across physiological signal types, manual preprocessing was inefficient, particularly for large‐scale multimodal datasets. To address this, BrainFusion provides automatic preprocessing pipelines customized for each signal type, with workflows that have been validated in previous studies and experimental applications.^[^ ^44^, ^45^, ^46^, ^47^ ^]^ The preprocessing steps, detailed in Figure S2 (Supporting Information), standardize signal cleaning and transformation, enhance processing efficiency, and ensure consistency and reproducibility across analyses.

#### 2.2.3 Cross‐Domain Feature Engineering

BrainFusion provides a comprehensive set of feature engineering methods, covering the time domain, frequency domain, time‐frequency domain, nonlinear, and network‐based analyses. These methods have been validated in previous studies, supporting their effectiveness across a range of multimodal brain‒body interaction analyses. Beyond these general approaches, BrainFusion also incorporates modality‐specific feature extraction techniques tailored to the distinct physiological characteristics of different signal types. For instance, the platform supports EEG microstate analysis, aperiodic component (1/f) modeling, EMG synergy decomposition, and ECG heart rate variability (HRV) analysis, among others. These specialized methods facilitate the extraction of physiologically relevant features, thereby enhancing the integrative analysis of multimodal signals. A detailed summary of the built‐in feature engineering functions was provided in Table S2 (Supporting Information).

#### 2.2.4 Multimodal Dynamic Coupling Analysis Engine

**Figure 2:**
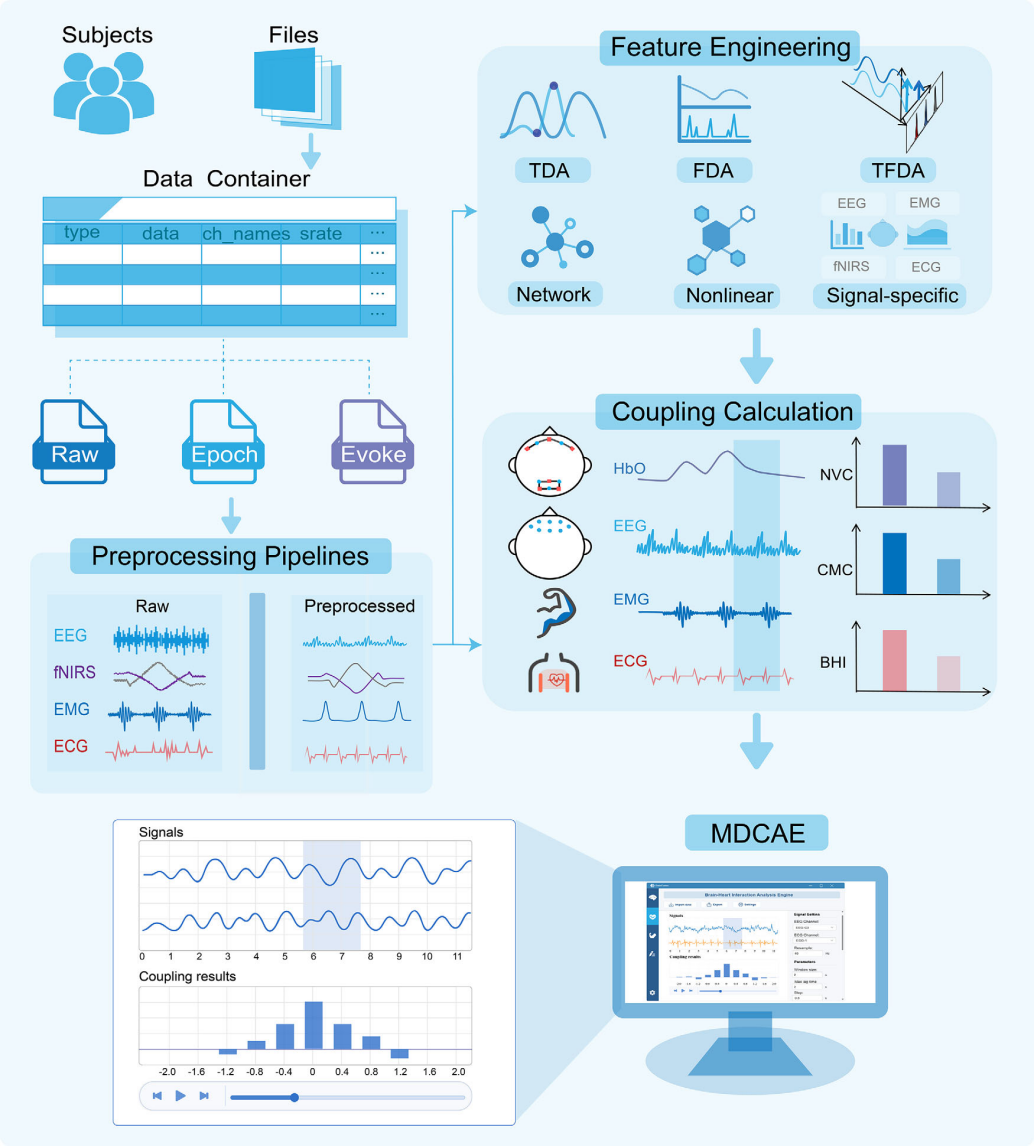
Multimodal physiological signal processing workflow in BrainFusion. Raw data from EEG, fNIRS, EMG, and ECG are organized in a standardized data container. Signals undergo modality‐specific preprocessing, followed by feature extraction across multiple analytical domains through time‐domain (TDA), frequency‐domain (FDA), and time‐frequency (TFDA) analysis, along with network and nonlinear methods. Dynamic coupling metrics such as NVC, CMC, and HBI are calculated and visualized through the multimodal dynamic coupling analysis engine (MDCAE).

BrainFusion also provides an intuitive experimental platform for the dynamic coupling analysis of multimodal BCI tasks and brain‒body interactions. By visualizing real‐time changes in intersignal relationships, BrainFusion enables the capture and quantification of dynamic coupling mechanisms under different experimental states, such as task and rest conditions. Predefined analysis modules were available for neurovascular coupling (NVC),^[^ ^48^, ^49^, ^50^, ^51^ ^]^ heart‒brain interaction (HBI),^[^ ^52^, ^53^, ^54^ ^]^ and corticomuscular coherence (CMC),^[^ ^55^, ^56^ ^]^ alongside customizable interfaces for implementing novel coupling metrics. Detailed descriptions of the definitions and computational procedures for NVC, CMC, and HBI were provided in the Supporting Information. The complete multimodal physiological signal processing workflow, from raw data import to coupling analysis and visualization, was illustrated in **Figure** **2**.

### 2.3 One‐Click Workflow Designer

**Figure 3:**
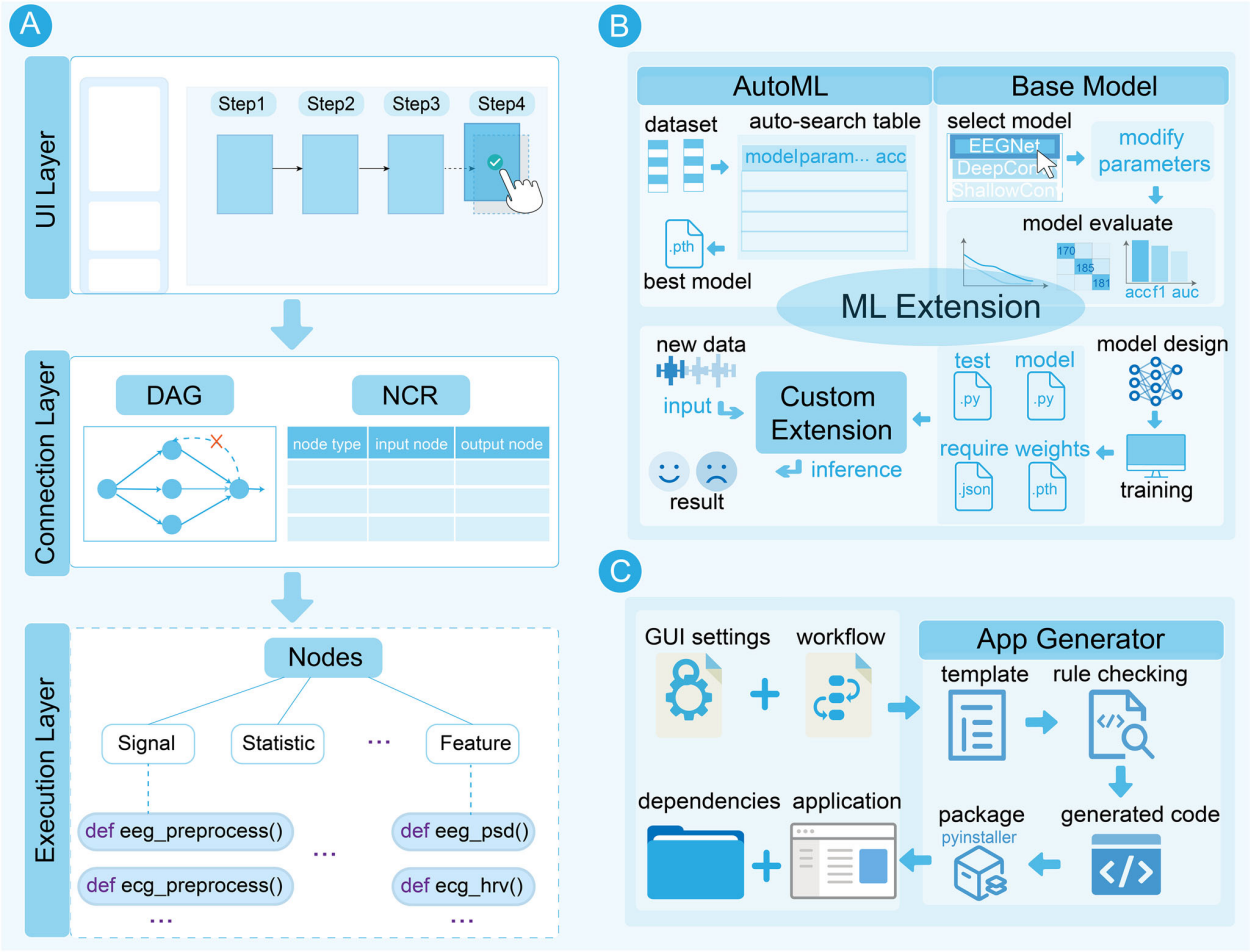
Illustration of the three major functional modules in BrainFusion. A) The one‐click workflow designer includes the UI layer for drag‐and‐drop workflow configuration, the connection layer for ensuring DAG structure compliance and node connection validation, and the execution layer comprising modular functional nodes. B) The machine learning extension framework supports traditional AutoML‐based model selection, deep learning benchmarking, and integration of user‐defined models via the external model interface. C) The application generator converts workflows into standalone executable applications with a GUI, enabling rapid deployment and demonstration.

The one‐click workflow designer in BrainFusion provides a drag‐and‐drop graphical user interface (GUI) for constructing complete workflows from data preprocessing to result analysis. Its architecture was organized into three distinct layers: the execution layer, the connection layer, and the UI layer. Functions within the execution layer were encapsulated as modular nodes, while the connection layer enforces rules to regulate node interactions. Together, these layers enable flexible and efficient workflow assembly within the UI layer (**Figure** **3**A).

#### 2.3.1 Execution Layer

BrainFusion adopts a modular node design, where each node encapsulates an independent function. These functions were categorized into eight types:

1. Data input: Reading various physiological signals, either individually or in batches.
2. Preprocessing: Performing standardized preprocessing on raw physiological signal data, with batch processing support.
3. Feature extraction: Converting raw signals into discrete features for subsequent analysis.
4. Coupling computations: Extracting coupling features between two or more physiological signals.
5. Machine learning: Including both model training and inference operations.
6. Statistical analysis: Comparing two or more datasets, including support for multiple comparison corrections.
7. Plotting: Visualizing input data without modifying its content.
8. General functions: Utility operations such as normalization, channel selection, and resampling.

Nodes were further categorized based on modality and input/output formats. For example, feature extraction nodes differ in output structure depending on whether they perform frequency‐domain or time‐domain analysis. Specialized preprocessing nodes, such as ECG‐specific preprocessing, were separately encapsulated to address modality‐specific constraints. A detailed classification of node types and their functionalities was provided in Table S3 (Supporting Information).

#### 2.3.2 Connection Layer

The connection layer ensures the logical correctness of node interactions by enforcing two primary rules:

1. Directed Acyclic Graph (DAG) Rule: All workflows must adhere to a DAG structure to prevent circular dependencies and deadlocks.
2. Node Connection Rule (NCR) Table: This table specifies valid input/output pairings between node types. For instance, a workflow must begin with a data input node, and an EEG preprocessing node must receive an EEG data node as input. The full set of connection rules was detailed in Table S3.

#### 2.3.3 UI Layer

The UI layer provides an intuitive GUI for visual workflow construction. Users can drag and drop nodes to configure workflows, monitor real‐time data flow, edit node parameters interactively, and validate workflow topologies against connection rules. Built‐in validation tools automatically detect circular dependencies or interface mismatches, thereby significantly lowering the technical barrier for users without programming experience.

#### 2.3.4 Debugging and Verification

BrainFusion supports basic debugging functionalities to ensure workflow correctness prior to deployment. During debugging, the platform generates synthetic input data matching the expected structure and executes each node sequentially. Logs record node execution status, and workflows that were complete without errors were deemed preliminarily verified and ready for real data application.

### 2.4 Machine Learning Extension Framework

The machine learning extension framework in BrainFusion was designed to enhance modeling efficiency for multimodal BCI research. It supports automated machine learning (AutoML)^[^ ^57^ ^]^ for traditional machine learning tasks and provides a baseline model library for deep learning applications. Furthermore, it enables the integration of externally developed models, ensuring flexibility for diverse experimental and translational needs (Figure 3B).

#### 2.4.1 Machine Learning Support

BrainFusion supports a wide range of traditional machine learning algorithms, including support vector machines (SVM), random forests (RF), and XGBoost. In addition, it integrates an AutoML system based on auto‐sklearn, which automates the search for optimal model architectures and hyperparameters. Users can define optimization objectives, such as maximizing classification accuracy or minimizing mean squared error, and BrainFusion automatically returns the best‐performing model configuration.

#### 2.4.2 Deep Learning Benchmark Model Library

To facilitate deep learning research, BrainFusion provides a dedicated baseline model library tailored for BCI applications. The library includes widely adopted architectures such as EEGNet,^[^ ^58^ ^]^ featuring predefined configurations and tunable parameters. This framework accelerates experimental benchmarking and improves reproducibility by offering standardized model implementations. At present, the integrated baseline models were primarily designed for two representative tasks: motor imagery classification and sleep stage classification. The model repository was actively being expanded to accommodate additional application scenarios and user requirements. Selected sleep‐stage classification models from this library were demonstrated in the case study presented in Section 3.2.

#### 2.4.3 External Model Interface

BrainFusion also offers a Python‐based class for integrating custom deep learning models via the external model interface. Currently, only models built with PyTorch were supported. Users were advised to maintain version consistency with the BrainFusion environment to avoid conflicts. For model inference, users need to provide the following files:

- A model weight file (.pth),
- A model definition file (.py),
- An inference function code file (.py),
- A model description file (.json), including model name, description, and data input/output formats.

By registering these components, BrainFusion enables the deployment and inference of custom models directly within its platform environment. Users were advised to ensure consistency between the BrainFusion system and external library versions to avoid compatibility issues.

### 2.5 Application Generator

BrainFusion includes an application generator module that transforms validated workflows into standalone executable applications (.exe files) featuring integrated GUIs. This process was designed to streamline prototype deployment and enhance translational applications (Figure 3C).

Application generation involves two primary stages:

1. UI Configuration: Users specify input data names, output interpretations, and application naming.
2. Computational Configuration: Users submit a validated workflow that includes a pretrained model for inference and output generation.

The system injects these parameters into a predefined code template, followed by rule‐based validation checks for completeness, logical consistency, and component compatibility. Upon passing validation, BrainFusion generates a final application script, which was then compiled via PyInstaller into a standalone executable, complete with all necessary libraries and dependencies. This ensures that the resulting application can operate independently on compatible systems without additional setup requirements.

## 3 Results

To systematically evaluate the capabilities of BrainFusion in multimodal neural signal processing, machine learning modeling, and rapid application deployment, two representative experimental scenarios were conducted. The first scenario involved a motor imagery classification task using a publicly available EEG‒fNIRS dataset, demonstrating the complete workflow of the platform, including multimodal preprocessing, feature extraction, NVC analysis, statistical evaluation, and classification modeling. The second scenario focused on automatic sleep stage classification based on EEG and ECG signals. In this case, a recently proposed deep learning model was reproduced and validated, followed by comparative benchmarking against baseline architectures. Subsequently, the model was deployed using the application generation module to create a functional prototype system. These two case studies collectively illustrate the capacity of BrainFusion to support comprehensive pipelines from data analysis and model training to the development of executable applications.

### 3.1 Case 1: EEG‐fNIRS Motor Imagery BCI Classification

This case study evaluated multimodal neural signal processing capabilities using a publicly available motor imagery BCI dataset from the Technical University of Berlin (TUB),^[^ ^59^ ^]^ comprising simultaneous EEG and fNIRS recordings during left‐hand, right‐hand motor imagery, and rest conditions. EEG signals were recorded via a 30‐channel BrainAmp system at 1000 Hz, and fNIRS data were acquired using a 36‐channel NIRScout system targeting motor cortical areas. The dataset featured a standard motor imagery paradigm with complementary spatiotemporal properties, making it well‐suited for multimodal analysis and studies of NVC.

**Figure 4:**
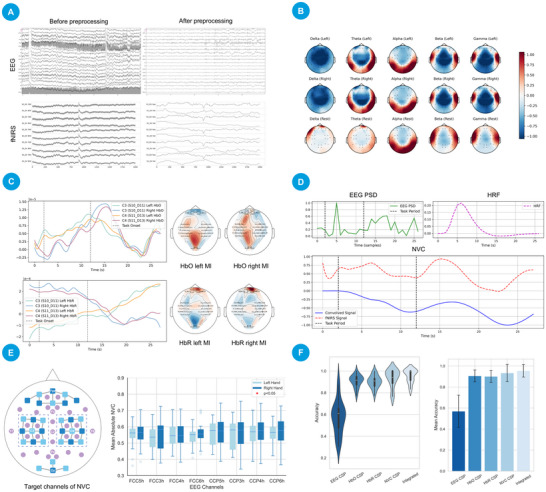
Analysis of the TU‐Berlin BCI dataset via BrainFusion. A) Comparison of EEG and fNIRS data before and after preprocessing. B) Topographic maps of EEG frequency bands (δ, θ, α, β, γ) during left‐handed and right‐handed motor imagery, and rest. C) The fNIRS channel layout corresponds to the motor cortex (C3/C4 regions), with average HbO and HbR responses extracted for motor‐related hemodynamic analysis. D) Neurovascular coupling modeling: EEG‐derived PSD (C3) convolved with a canonical HRF to predict fNIRS HbO signals. E) Left: Channel layout showing selected EEG electrodes and fNIRS channels. Right: Comparison of static NVC strengths between left and right‐hand motor tasks across EEG channels. Box plots show the distribution of mean absolute NVC values (range: 0–1) for each channel under left‐hand (light blue) and right‐hand (dark blue) conditions. Data represent 870 paired samples. No statistically significant differences were observed between the two conditions for any channel (two‐tailed paired t‐test, p < 0.05). F) Motor imagery classification performance. Violin plots showing the per‐subject mean 10‐fold cross‐validation accuracies (distribution), and bar plots showing the group‐level mean accuracies for EEG CSP (0.570 ± 0.152), HbO CSP (0.906 ± 0.047), HbR CSP (0.901± 0.052), NVC CSP (0.934 ± 0.067), and the ensemble model (0.955 ± 0.060).

To ensure compatibility across analytical pipelines, the original. mat format data were converted into a unified structure and standardized into BDF (for EEG) and SNIRF (for fNIRS) formats. During preprocessing, EEG signals were bandpass filtered between 2‒50 Hz to remove drift and high‐frequency noise, and notch filtered at 60 Hz to suppress power line interference. Ocular artifacts were eliminated using EOG regression followed by independent component analysis (ICA), and signals were subsequently re‐referenced to the average. For fNIRS, changes in oxygenated (HbO) and deoxygenated hemoglobin (HbR) concentrations were calculated using the modified Beer‐Lambert law. Noise and baseline drifts were mitigated by applying wavelet filtering (db4, four levels) and bandpass filtering (0.01‒0.7 Hz), and abnormal segments were corrected via spline interpolation, as shown in **Figure** **4**A.

In the feature extraction stage, EEG signals were decomposed into five canonical frequency bands—delta (0.5‒4 Hz), theta (4‒8 Hz), alpha (8‒13 Hz), beta (13‒30 Hz), and gamma (>30 Hz)—to compute power spectral density (PSD) features and generate topographic maps reflecting task‐specific cortical activations (Shown in Figure 4B). For fNIRS, channels positioned over C3 and C4 were selected to extract average HbO and HbR responses associated with motor cortical activation (Shown in Figure 4C).

To model neurovascular coupling, the average PSD curve of the C3 EEG electrode was convolved with a canonical hemodynamic response function (HRF) to generate predicted HbO trajectories, which were then compared to the measured fNIRS data (Shown in Figure 4D). Task‐related NVC differences were further quantified by extracting features from EEG C3/C4 electrodes and spatially corresponding fNIRS channels (Shown in Figure 4E), followed by paired t‐tests (conducted using SciPy v1.10, p < 0.05). The analysis included data from 29 participants, each completing three sessions of motor imagery tasks with 10 left‐hand and 10 right‐hand trials per session, resulting in a total sample size of n = 870 (29 × 3 × 10 × 2 conditions). The distributions of NVC strengths are visualized via box plots in Figure 4E, indicating no significant differences in NVC between the left‐ and right‐hand tasks (p ≥ 0.05 for all channels). This suggests that static NVC, as measured by EEG‐fNIRS coupling, does not exhibit task‐specific lateralization in the motor cortex during simple hand movement execution. To further investigate whether task‐specific patterns are embedded in the temporal dynamics of NVC, we next employed machine learning based on dynamic NVC features.

Subsequently, multimodal features were utilized for classification modeling. Common spatial pattern (CSP) features were extracted separately from EEG signals, HbO and HbR concentrations, and NVC time series. For each participant, SVM and RF classifiers were trained to distinguish between left‐hand and right‐hand motor imagery trials, with hyperparameters optimized via AutoML. The best‐performing model for each modality (EEG, HbO, HbR, NVC) was selected based on 10‐fold cross‐validation accuracy. These models were then integrated into a stacking ensemble framework, employing a linear SVM as the meta‐classifier to combine predictions from base learners.

As shown in Figure 4F, the classification results revealed average accuracies (mean ± standard deviation across participants) of 57.0% ± 15.2% for EEG CSP, 90.6% ± 4.7% for HbO CSP, 90.1% ± 5.2% for HbR CSP, and 93.4% ± 6.7% for NVC CSP features. The ensemble model achieved the highest overall accuracy of 95.5% ± 6.0%, demonstrating the substantial benefit of fusing spatiotemporal EEG features, hemodynamic responses, and NVC metrics for enhancing motor imagery BCI performance. These findings suggest that dynamic NVC may capture task‐specific neural‐hemodynamic interactions. A similar observation was recently reported in a vision‐related study, where dynamic NVC features were found to correlate with visual performance metrics.^[^ ^90^ ^]^

### 3.2 Case 2: Sleep Stage Classification and Application Generation

This case study was conducted to evaluate the capability of the BrainFusion platform in deep learning and application generation through an automatic sleep stage classification task. The Haaglanden Medisch Centrum (HMC) sleep staging database was utilized,^[^ ^60^ ^]^ comprising four‐channel EEG and single‐channel ECG signals, to establish a five‐class classification task targeting Wake, N1, N2, N3, and REM stages, based on the Rechtschaffen and Kales (R&K) sleep staging standard. Continuous EEG and ECG signals were segmented into 30‐second epochs and labeled accordingly using the data‐slicing module provided by the platform. Four deep learning models were compared: SleepNet^[^ ^61^ ^]^ (four‐channel EEG input), SleepEEGNet^[^ ^62^ ^]^ (single‐channel EEG input), AttnSleep^[^ ^63^ ^]^ (single‐channel EEG input), and CareSleepNet^[^ ^64^ ^]^ (four‐channel EEG combined with single‐channel ECG input). All models were trained under identical conditions, employing the Adam optimizer (initial learning rate of 1e‐4), cross‐entropy loss function, a batch size of 32, a maximum of 200 epochs, and early stopping if no improvement in validation loss was observed over 20 consecutive epochs. Five‐fold cross‐validation was applied, with 20% of the data reserved for the test set. For models utilizing a single EEG channel, results were reported based on the best‐performing channel.

**Figure 5:**
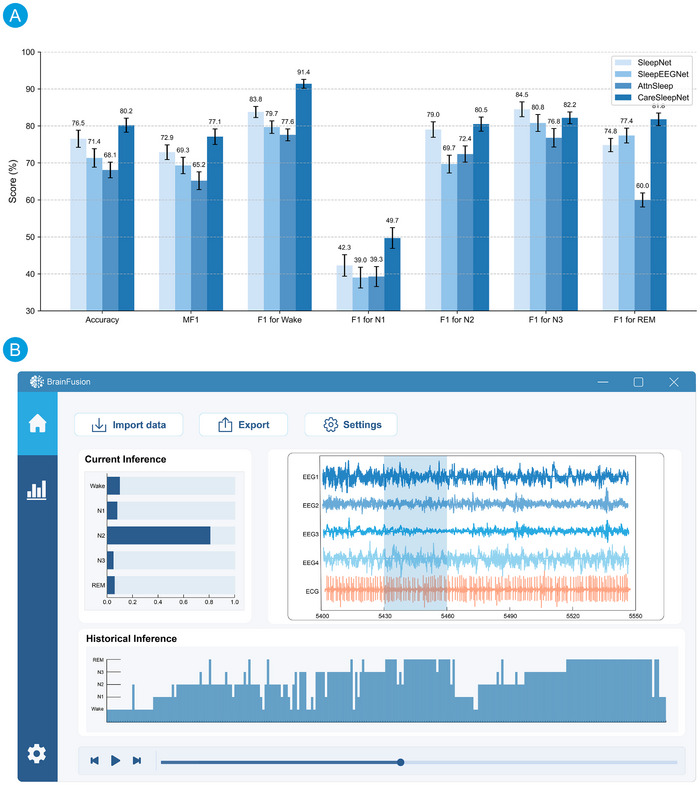
The results of sleep staging classification and generated application. A) Performance comparison of four deep learning models on the HMC dataset, presenting mean ± standard deviation (SD) for overall accuracy, macro‐averaged F1 score (mF1), and individual F1 scores across sleep stages (Wake, N1, N2, N3, REM). All metrics are averaged over 5‐fold cross‐validation. Error bars indicate one SD from the mean. B) User interface of the prototype inference application generated based on CareSleepNet, supporting real‐time sleep stage prediction, probability score visualization, and historical inference tracking.

The experimental results shown in **Figure** **5**A demonstrate that CareSleepNet achieved the best overall performance, with an accuracy of 80.2% ± 1.9 and a macro‐averaged F1 score (mF1) of 77.1% ± 2.1. In comparison, SleepNet reached an accuracy of 76.5% ± 2.3 and an mF1 of 72.9% ± 2.0, SleepEEGNet achieved 71.4% ± 2.5 accuracy and 69.3% ± 2.2 mF1, while AttnSleep attained 68.1% ± 2.1 accuracy and 65.2% ± 2.4 mF1.

For individual sleep stages, CareSleepNet also outperformed other models, with F1 scores of 91.4% ± 1.2 (Wake), 49.7% ± 2.8 (N1), 80.5% ± 1.9 (N2), 82.2% ± 1.6 (N3), and 81.8% ± 1.7 (REM). These results underscore the effectiveness of integrating multimodal EEG and ECG features for sleep stage classification.

After model training, CareSleepNet was imported into the BrainFusion platform through the external model interface, utilizing the provided model weights (.pth), architecture definition (.py), and inference description (.json). Furthermore, a prototype inference application was developed using the application generator module, as shown in Figure 5B, enabling the visualization of sleep stage predictions from EEG and ECG signal fragments, including classification labels, probability scores, and historical inference results. This process demonstrates the complete pipeline from model development to application deployment supported by the platform.

## 4 Discussion

The current ecosystem of neural signal analysis tools, including EEGLAB, FieldTrip, Brainstorm, and MNE‐Python, provides robust capabilities in specific domains such as single‐modality analysis, statistical modeling, and BCI construction. However, most existing tools offer limited support for end‐to‐end workflows that span multimodal integration, machine learning‐based modeling, and real‐world application deployment. BrainFusion aims to complement these tools by addressing key challenges in three interrelated dimensions:

1. Multimodal Signal Integration: While existing tools such as EEGLAB and BCILab^[^ ^65^ ^]^ are optimized for EEG‐centric analysis, BrainFusion facilitates unified processing of EEG, fNIRS, EMG, and ECG through standardized data containers and built‐in synchronization mechanisms. Its integrated coupling analysis framework enables systematic exploration of intermodality relationships. Brainstorm provides partial support for such analyses but lacks an integrated environment for multimodal feature fusion and workflow visualization.
2. Workflow Construction: Tools such as FieldTrip, MNE, BioSPPy,^[^ ^66^ ^]^ and NeuroKit2 require substantial coding effort, limiting accessibility for users without advanced programming skills. BrainFusion addresses this gap through a graphical workflow designer with modular node‐based architecture, supporting the intuitive assembly of processing pipelines. Its built‐in support for traditional machine learning and PyTorch‐based models further extends its applicability to modern AI‐driven analysis.
3. Translational Application Support: Most existing frameworks lack mechanisms for transforming research pipelines into deployable systems. Although OpenViBE enables real‐time BCI prototyping, its usage is primarily limited to experimental settings. BrainFusion introduces an automated application generation module that enables the packaging of analytical workflows into executable applications, thus facilitating translation from laboratory research to pilot studies and clinical trials (**Table** **1**).

**Table 1:** Functional comparison between BrainFusion and existing neural signal analysis tools.

Software	Interface	Supported Signals	Cross‐Modal Analysis	AI Support	Workflow Design	App Generation	Real‐Time Processing
EEGLAB^[^ ^13^ ^]^	GUI + MATLAB scripting	EEG	Limited	No	Limited	No	No
FieldTrip^[^ ^14^ ^]^	MATLAB scripting	EEG, MEG, ECoG	Yes	Limited	Yes	No	No
Brainstorm^[^ ^16^ ^]^	GUI + MATLAB scripting	EEG, MEG, fNIRS	Yes	Limited	Yes	No	No
SPM^[^ ^19^ ^]^	GUI + MATLAB scripting	fMRI, EEG, MEG	Yes	Limited	Yes	No	No
BCILab^[^ ^65^ ^]^	MATLAB scripting	EEG (BCI‐optimized)	No	Yes	Yes	No	Yes
MNE‐Python^[^ ^31^ ^]^	Python scripting	EEG, MEG, fNIRS	Yes	Limited	No	No	No
BioSPPy^[^ ^66^ ^]^	Python scripting	ECG, EDA, EMG	No	No	No	No	No
OpenViBE^[^ ^39^ ^]^	GUI + Lua/C++ scripting	EEG	Limited	Yes	Yes	No	Yes
Neurokit2^[^ ^30^ ^]^	Python scripting	ECG, EDA, EEG (basic)	No	No	No	No	No
**BrainFusion**	**GUI + Python scripting**	**EEG, fNIRS, EMG, ECG**	**Yes**	**Yes**	**Yes**	**Yes**	**No**

Note: “Yes” indicates native support; “Limited” denotes partial support through extensions or third‐party dependencies; “No” signifies unsupported functionality.

The brain, as the central component of the central nervous system (CNS), maintains intricate bidirectional communication with the peripheral nervous system (PNS).^[^ ^67^ ^]^ In recent years, electrophysiological signals have been increasingly utilized to investigate this interaction, particularly through BHI and CMC frameworks.^[^ ^68^, ^69^, ^70^ ^]^ BHI typically combines EEG and ECG to examine the coupling between brain activity and autonomic nervous system (ANS) responses, and has shown promise in emotion recognition,^[^ ^71^, ^72^ ^]^ BCI development,^[^ ^73^ ^]^ and disease monitoring.^[^ ^74^ ^]^ CMC, derived from EEG and EMG recordings, reflects the interplay between cortical motor commands and muscular responses. It has been applied in motor function evaluation post‐stroke, action classification, and differentiation of imagined versus executed movement, contributing to both neurorehabilitation and hybrid BCI systems.^[^ ^75^, ^76^, ^77^, ^78^, ^79^ ^]^

Beyond electrophysiological combinations, integrating hemodynamic signals such as fNIRS with EEG offers a complementary view of brain activity. While EEG captures fast neural oscillations, fNIRS reflects underlying hemodynamic responses governed by NVC.^[^ ^80^, ^81^ ^]^ Alterations in NVC have been associated with aging and neuropsychiatric conditions,^[^ ^82^ ^]^ and combining EEG with fNIRS has been shown to enhance NVC estimation by leveraging complementary temporal and spatial resolutions,^[^ ^83^ ^]^ and mitigating modeling biases present in conventional general linear models (GLMs).^[^ ^84^ ^]^ EEG–fNIRS integration has been explored in various cognitive and sensory tasks, including visual and auditory processing,^[^ ^85^, ^86^, ^87^, ^88^, ^89^, ^90^ ^]^ and has been proposed as a candidate modality for hybrid BCIs and clinical applications such as Alzheimer's disease diagnosis.^[^ ^91^, ^92^, ^93^ ^]^

In light of these diverse applications, a flexible and unified analysis platform is essential for advancing multimodal physiological signal research. BrainFusion aims to support such efforts by offering dynamic coupling analysis tools, modality‐specific preprocessing pipelines, and a cross‐domain feature extraction library. By facilitating workflows involving EEG‒fNIRS‐based NVC analysis, EEG‒ECG‐based BHI studies, and EEG–EMG‐based CMC evaluation, BrainFusion may contribute to a more integrated understanding of central and peripheral dynamics in both healthy and pathological states.

Although BrainFusion provides a unified and extensible platform for multimodal physiological signal analysis, several limitations warrant consideration. First, the current implementation is primarily designed for offline data analysis and relies on file‐based inputs, which constrains its applicability in real‐time applications such as closed‐loop BCI systems or online neurofeedback. While the modular architecture allows for future integration of real‐time data streaming, this functionality is not yet available.

Second, although the framework includes a set of widely used preprocessing and coupling analysis tools, it does not encompass all advanced or computationally demanding methods. Techniques such as deep learning‐based multimodal fusion or dynamic causal modeling, which are increasingly relevant in neuroscience, are currently supported to a limited extent and may require integration with external libraries or custom development.

Third, BrainFusion presently supports a core set of physiological modalities—namely EEG, ECG, EMG, and fNIRS. While these are representative of many brain–body interaction studies, the absence of support for additional signals such as EDA, magnetoencephalography (MEG), or respiratory metrics limits its applicability to broader experimental paradigms.

Several directions are envisaged for future development. Enhancing support for real‐time signal acquisition and online processing would enable closed‐loop and interactive applications. Expanding the model library to include baseline pipelines for domains such as cognitive workload estimation and affective computing could further broaden the framework's utility. In addition, the generation of customizable application templates for use cases like multimodal emotion recognition or stress monitoring may facilitate rapid prototyping. Further inclusion of advanced connectivity metrics, including nonlinear and directional coupling analyses, would improve support for dynamic systems modeling. Last, continued refinement of the graphical user interface and comprehensive documentation will be critical for enhancing accessibility, particularly for users with limited programming expertise.

## 5 Conclusion 

BrainFusion introduces a unified software framework that complements the existing software ecosystem for multimodal physiological signal analysis by integrating standardized data processing pipelines, cross‐domain feature engineering, and dynamic coupling computation within a modular, low‐code workflow architecture. The framework's efficacy is demonstrated through case studies involving motor imagery and sleep staging tasks, where its capacity to streamline multimodal integration and enhance classification robustness underscores the value of combining complementary neural and physiological signatures. By enabling graphical workflow design, automated machine learning benchmarking, and deployable application generation, BrainFusion bridges critical gaps between experimental research and translational prototyping while promoting reproducibility and accessibility. Future developments aim to expand real‐time processing capabilities, incorporate advanced analytical methods, and broaden modality support, positioning the framework as a foundational tool for advancing interdisciplinary studies of brain–body interactions and fostering user‐centered innovation in neurophysiological computing.

## Conflict of Interest

The authors declare no conflict of interest.

## Software and Code Availability

BrainFusion software and its entire code are available at https://github.com/lwh‐scut/BrainFusion.

## Supporting information



Supporting Information

## Data Availability

This study makes use of publicly available datasets. The EEG‐fNIRS motor imagery BCI dataset used in Case 1 is provided by the Technical University of Berlin^[^ ^59^ ^]^ and can be accessed at: https://doc.ml.tu‐berlin.de/hBCI/contactthanks.php The sleep staging dataset used in Case 2 is from the Haaglanden Medisch Centrum^[^ ^60^ ^]^ and is available via PhysioNet at: https://physionet.org/content/hmc‐sleep‐staging/1.1/
