# Supplementary material for: BrainFusion: a Low‐Code, Reproducible, and Deployable Software Framework for Multimodal Brain‒Computer Interface and Brain‒Body Interaction Research
Source: Adv Sci (Weinh). 2025 Jun 5;12(32):e17408. doi: 10.1002/advs.202417408 (PMC12407257; doi:10.1002/advs.202417408)
Supplement: Supplementary file 1 — Supporting Information [file ADVS-12-e17408-s001.docx]

Supporting Information

**BrainFusion: A Comprehensive Software for Multimodal Physiological Signal Analysis in Brain-Computer Interface and Neuroscience Research**

Wenhao Li , Chenyang Gao, Zhaobo Li, Yunheng Diao, Jiaxin Li, Jiayi Zhou, Jing Zhou, Ying Peng, Guanchu Chen, Xuechen Wu, Kai Wu*

BIDS Converter

The BIDS Converter in BrainFusion simplifies the conversion of EEG data into a BIDS-compliant format for easy sharing and analysis. As shown in **Figure S2**, users select the data file, input essential metadata (such as subject ID and task name), and generate standardized output files, including the raw EEG file, JSON metadata, channels, and events files. This ensures compatibility with data analysis tools and facilitates reproducible, shareable datasets.


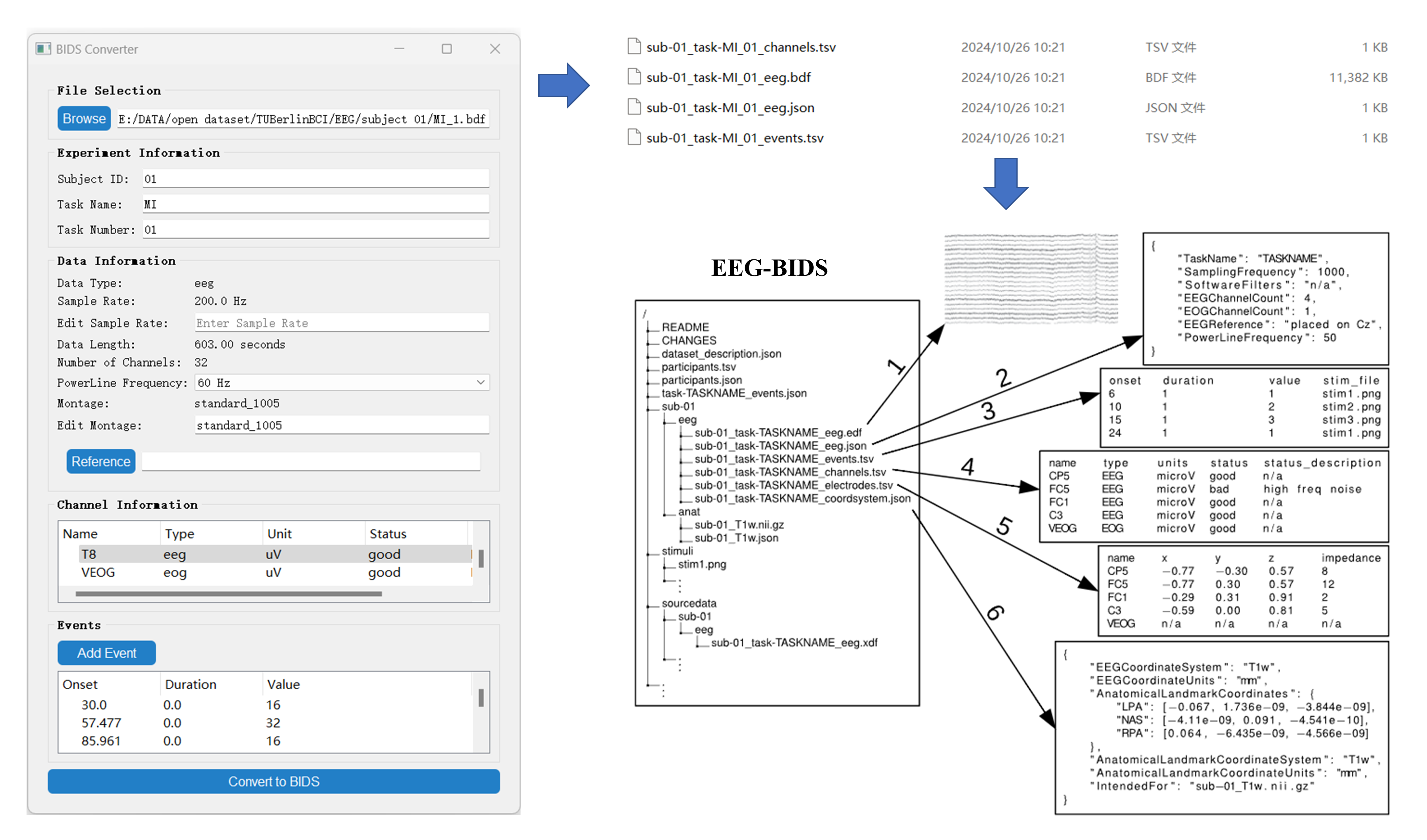


**Figure S1** EEG-BIDS Converter in BrainFusion.


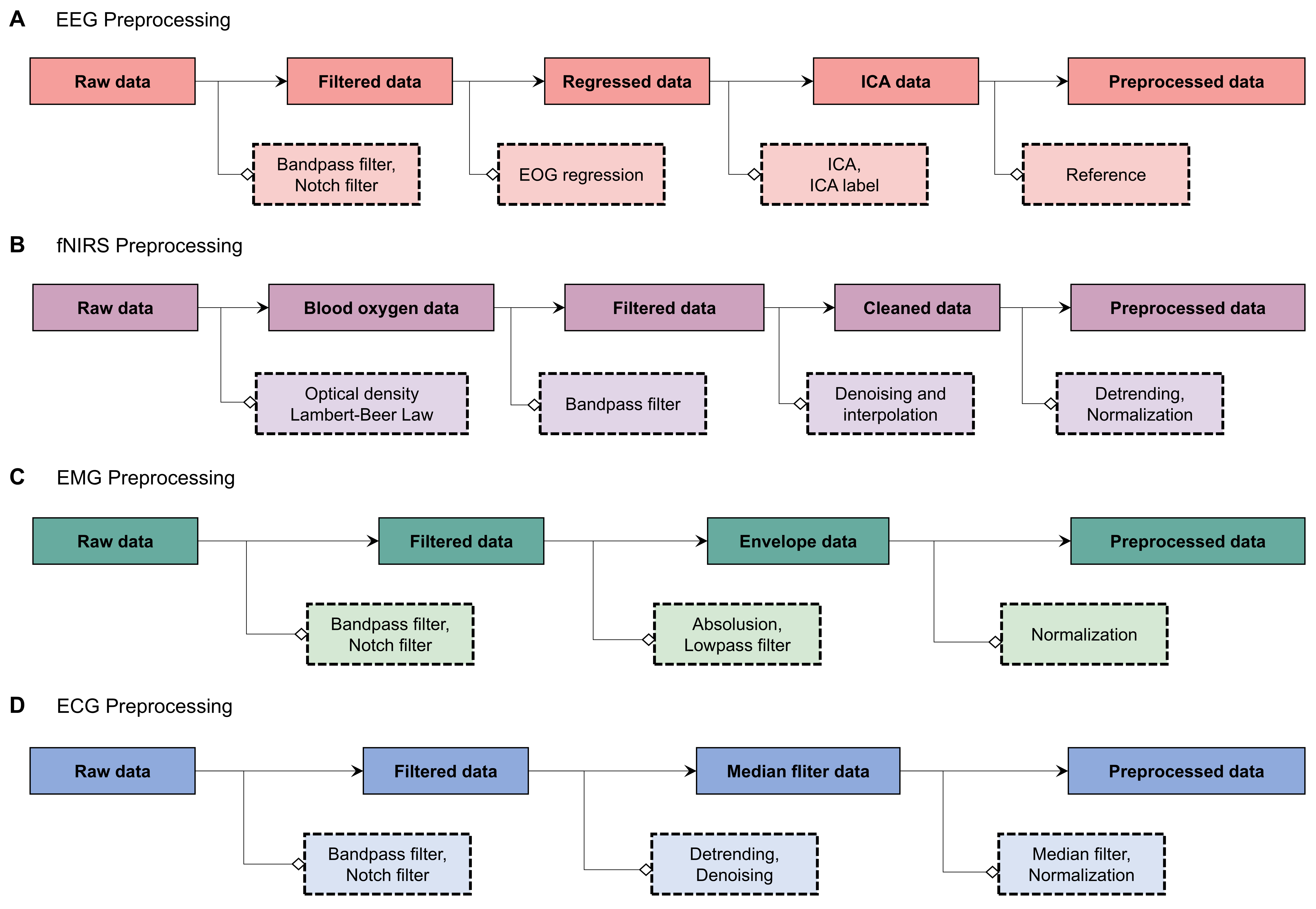


**Figure S2** Signal preprocessing pipelines in BrainFusion. (A) EEG preprocessing (0.5‒100 μV) employs notch filtering (50/60 Hz) and bandpass filtering (1‒100 Hz) to eliminate powerline interference and drifts; integrates EOG regression (optional) and ICA decomposition (Infomax/Picard/FastICA) with automated artifact removal via ICALabel (retaining only "brain" components); signals are re-referenced post-reconstruction (electrode-specific or average reference). (B) fNIRS processing converts optical densities to HbO/HbR via MBLL; applies bandpass filtering (0.01‒0.2 Hz), motion correction (wavelet), detrending, and normalization (z-score/mean subtraction) to suppress physiological noise and hemodynamic fluctuations. (C) EMG artifact removal combines notch filtering (50/60 Hz) and bandpass filtering (20‒450 Hz); rectifies signals via absolute conversion, smoothes with moving average/low-pass filter, and normalizes for cross-trial/subject consistency. (D) ECG preprocessing utilizes bandpass filtering (0.5‒40 Hz) with optional downsampling (250 Hz); and incorporates detrending, wavelet thresholding (artifact suppression), median filtering (200 ms window), and normalization to mitigate EMG/motion interference.

**Explanation of ICALabel**

To effectively separate neural signals from non-neural artifacts, BrainFusion incorporates independent component analysis (ICA) as a key preprocessing step. The platform supports multiple ICA algorithms, including Infomax, Picard, and FastICA, allowing users to select the most suitable decomposition method for their dataset. BrainFusion also provides flexibility in the number of independent components to retain, enabling users to define the number of extracted components based on their specific experimental needs. For artifact removal, BrainFusion employs the ICALabel framework^[44]^, which automatically classifies independent components into categories such as brain, ocular, muscular, cardiac, line noise, and other artifacts. By default, only components classified as 'brain' are retained, ensuring that non-neural sources of contamination are systematically removed without requiring manual intervention. This automated approach enhances reproducibility and minimizes subjective bias in artifact rejection.

Multimodal Physiological Coupling Pipelines

Multimodal physiological coupling refers to the interaction and synchronization between different physiological systems such as nerves, blood vessels, muscles and the heart. In BrainFusion, the coupling pipelines are designed to compute NVC, BHI and CMC. These pipelines simplify the process of physiological signal coupling computation and are essential for researchers to understand the integrated function of various physiological systems and their relevance in health and disease.

(1) Neurovascular coupling pipeline

NVC describes the relationship between neural activity and cerebral blood flow. In 2002, Cohen integrated EEG and fMRI to study NVC by convolving the PSD of EEG with the hemodynamic response function (HRF) and correlating it with fMRI data^[48,49]^. In 2020, Li extended this approach using EEG and fNIRS to increase task-induced brain region identification^[50]^. Chiarelli (2021) applied principal component analysis (PCA) to EEG and reported neurovascular decoupling in patients with Alzheimer's disease. Cross-frequency coupling (CFC) techniques, such as phase-amplitude and amplitude-amplitude coupling, have been explored for NVC in EEG-fMRI studies. Recent research has also focused on the directionality of NVC using methods like transfer entropy and Granger causality^[51]^. The NVC pipeline of BrainFusion preprocesses EEG and fNIRS data, offering multiple methods for calculating NVC, including GLM, CFC, and causality analysis. The platform supports both single and batch processing, allowing users to configure parameters and visualize results.

(2) Brain-Heart Interaction Pipeline

The BHI describes the relationship between brain activity and cardiac function, which is crucial for autonomic nervous system regulation. Typically, EEG and ECG are used to study how brain activity influences the heart and vice versa. In BHI studies, the LF/HF ratio of heart rate variability is commonly correlated with EEG power in specific frequency bands. This approach has been applied in epilepsy prediction^[52]^, sleep disorders^[53]^, and stress detection^[54]^. In BrainFusion, the BHI pipeline preprocesses EEG and ECG data, extracts features, and calculates coupling. For ECG, the Pan-Tompkins algorithm detects QRS complexes and R-peaks, generating HRV data and extracting LF/HF components. For EEG, power across frequency bands is calculated. BrainFusion supports several coupling methods, including Pearson correlation, coherence, cross-frequency coupling, and causality analysis.

(3) Cortico-Muscular Coupling Pipeline

The CMC examines the relationship between the motor cortex and muscle activity, which is essential for understanding motor control and disorders. In BrainFusion, the CMC pipeline preprocesses EEG and EMG data, and then analyzes functional connectivity through coherence analysis, which assesses the frequency-specific correlation between EEG and EMG signals. High coherence in the beta band indicates strong cortico-muscular coupling, which is crucial for fine motor control.^[55,56]^ Additionally, BrainFusion supports advanced analyses, such as Granger causality to determine directionality and wavelet coherence for time-resolved coupling measures. These methods are vital for studying motor command execution in voluntary and involuntary muscle responses.

| **Data Field** | Description | Format | Detail |
| --- | --- | --- | --- |
| **data** | Contains the sampled data for each channel | Array | Shape: number of channels × number of samples |
| **ch_names** | Channel names | List | Contains the names of each channel |
| **srate** | Sampling rate | Float or List | Representing the sampling rate |
| **events** | Event markers (timestamps) | Array or list | Each event marker is formatted as [event, time, duration] |
| **montage** | Electrode configuration | String | Standard electrode montage type for plotting and ICA |
| **loc** | Sensor 3D coordinates | Array | 3D coordinates of the sensors |
| **type** | Sensor type | String | Labeled as eeg/fnirs/emg/ecg, etc. |
| **sd** | [specific to NIRS] Marks the sources and receivers of the channels | Array or list | specific to NIRS |
| **wavelengths** | [specific to NIRS] Infrared light frequencies | Array or list | specific to NIRS |

Table S1. Structure of Standardized Data Container

| **Feature Type** | **Features** |
| --- | --- |
| Time | RMS, Variance, Mean Absolute Value, Zero Crossing Rate, Hjorth Parameters |
| Frequency | Center Frequency, PSD, Mean Frequency, Average Power |
| Time-frequency | STFT, Wavelet Transform, Wavelet Packet Energy |
| Nonlinear | Sample Entropy, Multiscale Entropy, Approximate Entropy, Fractal Dimension |
| Network | Node Degree, Clustering Coefficient, Centrality, Modularity, Average Shortest Path Length, Average Clustering Coefficient, Network Density |
| EEG-specific | Average Power of (δ,θ,α,β,γ) bands, Aperiodic Parameters, Microstate |
| ECG-specific | RR Intervals, P Wave, T Wave, Q Wave, Heart Rate Variability |
| EMG-specific | IEMG, Muscle Synergy |
| fNIRS-specific | Average HbO, Average HbR, Average HbT |

**Table S2.** **Feature extraction framework for multimodal biosignals in BrainFusion**

Note: This table summarizes the feature engineering pipelines across electrophysiological (EEG/ECG/EMG) and hemodynamic (fNIRS) modalities. Abbreviations: RMS (Root Mean Square), PSD (Power Spectral Density), STFT (Short-Time Fourier Transform), HbO (oxyhemoglobin), HbR (deoxyhemoglobin), HbT (total hemoglobin), IEMG (Integrated Electromyography). Frequency bands for EEG: δ (0.5‒4 Hz), θ (4‒8 Hz), α (8‒12 Hz), β (12‒30 Hz), γ (>30 Hz). All features are computed from preprocessed signals using platform-optimized algorithms.

| Node Type | Node Description | Input Nodes | Output Nodes |
| --- | --- | --- | --- |
| EEG_SIN | Read a single EEG file as raw input | None | EEG_PRE, FE_TD, FE_FD, EEG_PSD, EEG_NPA, EEG_MS, FE_TFD, FE_NL, FE_NET_L, FE_NET_G |
| ECG_SIN | Read a single ECG file | None | ECG_PRE, ECG_RR, ECG_PTQ, FE_TD, FE_FD, FE_TFD, FE_NL |
| EMG_SIN | Read a single EMG file | None | EMG_PRE, EMG_IEMG, EMG_MSYN |
| FNIRS_SIN | Read a single fNIRS file | None | FNIRS_PRE, FNIRS_HBO |
| EEG_BIN | Batch import EEG data | None | EEG_PRE, FE_TD, FE_FD, EEG_PSD, EEG_NPA, EEG_MS |
| ECG_BIN | Batch import ECG data | None | ECG_PRE, ECG_RR, ECG_PTQ |
| EMG_BIN | Batch import EMG data | None | EMG_PRE, EMG_IEMG, EMG_MSYN |
| FNIRS_BIN | Batch import fNIRS data | None | FNIRS_PRE, FNIRS_HBO |
| EEG_PRE | EEG preprocessing | EEG_SIN, EEG_BIN | FE_TD, FE_FD, FE_TFD, FE_NL, EEG_PSD, EEG_MS, EEG_NPA, CMC, NVC, HBI |
| ECG_PRE | ECG preprocessing | ECG_SIN, ECG_BIN | ECG_RR, ECG_PTQ, ECG_HRV, HBI |
| EMG_PRE | EMG preprocessing | EMG_SIN, EMG_BIN | EMG_IEMG, EMG_MSYN, CMC |
| FNIRS_PRE | fNIRS preprocessing | FNIRS_SIN, FNIRS_BIN | FNIRS_HBO, NVC |
| FE_TD | Extract time-domain features | EEG_PRE, ECG_PRE, EMG_PRE, FNIRS_PRE, FUNC_TS | STAT, ML_TRN, ML_INF, PLOT_BAR, PLOT_SCAT, FUNC_DS |
| FE_FD | Extract frequency-domain features | EEG_PRE, ECG_PRE, EMG_PRE, FNIRS_PRE, FUNC_TS | PLOT_LINE, PLOT_BAR, PLOT_HEAT, FUNC_DS |
| FE_TFD | Time-frequency transforms like STFT, wavelet | EEG_PRE, ECG_PRE, EMG_PRE, FNIRS_PRE, FUNC_TS | PLOT_LINE, PLOT_HEAT, FUNC_DS |
| FE_NL | Nonlinear features like entropy, complexity | EEG_PRE, ECG_PRE, EMG_PRE, FNIRS_PRE, FUNC_TS | PLOT_BAR, ML_TRN, FUNC_DS |
| FE_NET_L | Local network features | EEG_PSD, FE_FD | STAT, FUNC_DS |
| FE_NET_G | Global network features | EEG_PSD, FE_FD | FUNC_DS |
| EEG_PSD | Compute EEG Power Spectral Density | EEG_PRE | PLOT_HEAT, PLOT_TOPO, FUNC_DS |
| EEG_NPA | EEG non-parametric analysis | EEG_PRE, EEG_PSD | STAT |
| EEG_MS | EEG microstate features | EEG_PRE | FUNC_DS, STAT, PLOT_BAR |
| ECG_RR | Extract RR intervals from ECG | ECG_PRE | ECG_HRV |
| ECG_PTQ | Extract P, T, and Q wave features | ECG_PRE | ECG_HRV |
| ECG_HRV | Heart Rate Variability analysis | ECG_RR, ECG_PTQ | FUNC_DS, PLOT_BAR, ML_TRN |
| EMG_IEMG | Integrated EMG analysis | EMG_PRE | FUNC_DS, PLOT_BAR |
| EMG_MSYN | EMG muscle synergy features | EMG_PRE | CMC, FUNC_DS |
| FNIRS_HBO | Separate HbO and HbR from fNIRS | FNIRS_PRE | NVC, FUNC_DS |
| NVC | Neurovascular coupling analysis (EEG + fNIRS) | EEG_PRE, FNIRS_HBO | FUNC_DS, PLOT_HEAT |
| CMC | Cortico-Muscular Coupling (EEG + EMG) | EEG_PRE, EMG_MSYN | FUNC_DS |
| HBI | Heart-Brain Interaction (EEG + ECG) | EEG_PRE, ECG_PRE | FUNC_DS, STAT |
| CCPL | Custom coupling analysis (e.g., Granger, MI) | EEG_PRE, ECG_PRE, EMG_PRE, FNIRS_PRE | FUNC_DS, PLOT_HEAT |
| STAT | Statistical tests like t-tests, ANOVA | FE_TD, FE_FD, FE_NL, EEG_NPA, EEG_MS, HBI | FUNC_DS, PLOT_BAR |
| ML_TRN | Train machine learning model (SVM, RF, etc.) | FUNC_DS | ML_INF |
| ML_INF | Predict with a trained machine learning model | FUNC_DS | FUNC_DS |
| DL_TRN | Train deep neural networks | EEG_PRE, FE_TD, FE_FD, FUNC_DS | DL_INF |
| DL_INF | Predict using deep learning models | EEG_PRE, FUNC_DS | FUNC_DS |
| PLOT_LINE | Plot line graphs for time-series | EEG_PRE, FE_FD, FE_TFD | None |
| PLOT_BAR | Plot bar graphs | FUNC_DS, STAT | None |
| PLOT_HEAT | Plot heatmaps | EEG_PSD, NVC, CCPL | None |
| PLOT_SCAT | Plot scatter graphs | FUNC_DS | None |
| PLOT_TOPO | Topographic brain maps | EEG_PSD | None |
| FUNC_TS | Time-series utilities (normalize, resample, etc.) | EEG_PRE, ECG_PRE, EMG_PRE, FNIRS_PRE | EEG_PRE, ECG_PRE, EMG_PRE, FNIRS_PRE |
| FUNC_DS | Discrete data utilities (normalize, encode, etc.) | FE_TD, FE_FD, EEG_MS, ECG_HRV, ML_INF | ML_TRN, DL_TRN, STAT |
| FUNC_MTX | Matrix utilities (threshold, symmetry, etc.) | EEG_PSD, CCPL, FE_NET_L, FE_NET_G | PLOT_HEAT |

**Table S3. The table of node connection rules**

Note: All nodes are denoted using standardized English abbreviations to ensure a concise representation of the table content. The detailed node names are provided below.

EEG_SIN：Single EEG Input Node

ECG_SIN：Single ECG Input Node

EMG_SIN：Single EMG Input Node

FNIRS_SIN：Single fNIRS Input Node

EEG_BIN：Batch EEG Input Node

ECG_BIN：Batch ECG Input Node

EMG_BIN：Batch EMG Input Node

FNIRS_BIN：Batch fNIRS Input Node

EEG_PRE：EEG Preprocessing Node

ECG_PRE：ECG Preprocessing Node

EMG_PRE：EMG Preprocessing Node

FNIRS_PRE：fNIRS Preprocessing Node

FE_TD：Time Domain Feature Extraction Node

FE_FD：Frequency Domain Feature Extraction Node

FE_TFD：Time-Frequency Feature Extraction Node

FE_NL：Nonlinear Feature Extraction Node

FE_NET_L：Local Network Feature Node

FE_NET_G：Global Network Feature Node

EEG_PSD：EEG Power Spectral Density Feature Node

EEG_NPA：EEG Non-parametric Analysis Node

EEG_MS：EEG Microstate Feature Node

ECG_RR：ECG RR Interval Node

ECG_PTQ：ECG P-T-Q Wave Feature Node

ECG_HRV：ECG Heart Rate Variability Node

EMG_IEMG：EMG Integrated EMG Node

EMG_MSYN：EMG Muscle Synergy Node

FNIRS_HBO：fNIRS HbO/HbR Extraction Node

NVC：Neurovascular Coupling Node

CMC：Cortico-Muscular Coupling Node

HBI：Heart-Brain Interaction Node

CCPL：Custom Coupling Analysis Node

STAT：Statistical Analysis Node

ML_TRN：Machine Learning Training Node

ML_INF：Machine Learning Inference Node

DL_TRN：Deep Learning Training Node

DL_INF：Deep Learning Inference Node

PLOT_LINE：Line Plot Node

PLOT_BAR：Bar Plot Node

PLOT_HEAT：Heatmap Plot Node

PLOT_SCAT：Scatter Plot Node

PLOT_TOPO：Topographical Brain Map Plot Node

FUNC_TS：Generic Time-Series Function Node

FUNC_DS：Generic Discrete Data Function Node

FUNC_MTX：Generic Matrix Data Function Node
